# Role of antibody-based therapy in indolent non-Hodgkin's lymphoma

**DOI:** 10.1016/j.lrr.2021.100275

**Published:** 2021-10-24

**Authors:** Patrick Willard, John McKay, Victor Yazbeck

**Affiliations:** a Virginia Commonwealth University, Department of Internal Medicine, Richmond, VA, United States of America; b Wake Forest Baptist Comprehensive Cancer Center, Winston-Salem, NC, United States of America; c Virginia Commonwealth University, Massey Cancer Center, Richmond, VA, United States of America

**Keywords:** Indolent non-Hodgkin lymphoma, Immunotherapies, Monoclonal antibodies, Antibody-drug conjugate, bispecific antibodies, treatment, bispecific antibodies, treatment

## Abstract

Monoclonal antibodies (mAb) for indolent non-Hodgkin's lymphoma (iNHL) including follicular and marginal zone lymphomas was a key therapeutic development that changed the natural history of these diseases. Rituximab, a chimeric anti-CD20 mAb, was the first immunotherapy ever used in cancer, and a current cornerstone of lymphoma therapies. Since, we saw development of humanized antibodies, next generations anti-CD20, mAbs targeting other markers on tumor cells (CD19 and CD22), its microenvironment (PD-1, CD47), antibody drug conjugates and bispecific T cell engagers. Given their activity, safety and specificity, mAbs are well poised to remain an essential therapeutic tool for iNHL and other malignancies.

## 1 Introduction

**Fig. 1:**
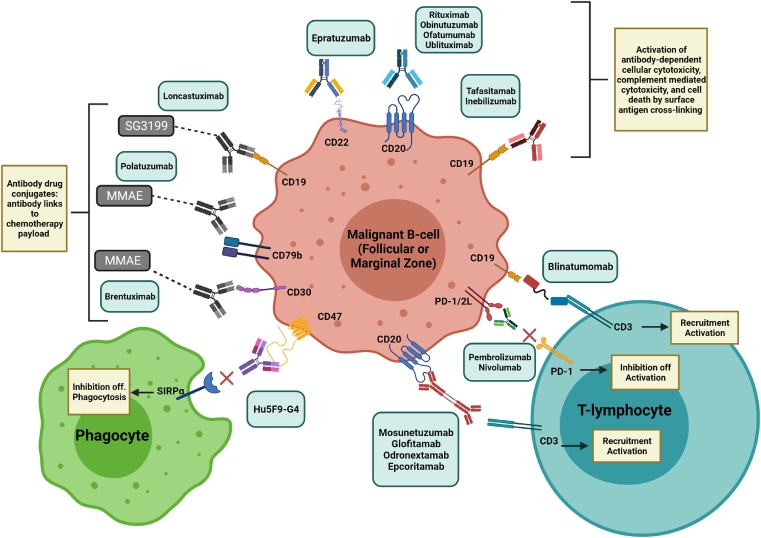
The effects of various mAb that target cell surface proteins with variable responses by host immune system. MMAE: Monomethyl auristatin E. Created with BioRender.com. Publishing rights granted.

Research efforts involving indolent non-Hodgkin's lymphoma (iNHL) that includes follicular lymphoma (FL) and marginal zone lymphoma (MZL), like most hematologic and solid malignancies, have been centered on therapies incorporating monoclonal antibodies (mAbs). Given their enhanced specificity, these antibodies target specific markers on cancer cells by activating the patient's own immune system via several mechanisms leading to cancer cell death without the widespread, non-specific cytotoxic effect of conventional chemotherapies (Fig. 1). The use of mAbs originates back to 1955 when Niels Jerne postulated the “natural selection theory” of antibody formation. He proposed that an entire range of possible antibody specificities were produced and circulated in serum, which bind to specific antigens that will lead to replication of a specific antibody, initiating humoral immunity. Prior to this, Cesar Milstein developed transformed B cells that produced unregulated amounts of nonspecific antibodies. Influenced by Jerne's idea of antibody production, George Kohler immunized a mouse with sheep red blood cells, collected splenic B cells and fused them with Milstein's myeloma cell, leading to the development of the hybridoma technique for the eternal production of monoclonal antibodies with predetermined specificity using cell cultures. Based on this work, the three researchers shared the 1984 Nobel Prize in Physiology or Medicine [1].

Monoclonal antibodies development was initially focused on treating acute transplant rejection with Orthoclone OKT3 (muromonab-CD3) being the first fully murine derived mAb to receive FDA approval back in 1986. In order to reduce the anti-species immune response that can be seen with the murine type, chimeric mAbs were developed: they consist of murine variable regions and the human constant regions. Abciximab, was the first chimeric type mAB to be developed as an antiplatelet agent in 1994 [2].

In 1985, George Smith developed phage display, where a bacteriophage, a virus that infects bacteria, can be used to evolve new proteins. Using phage display, Gregory Winter directed the evolution of antibodies toward the production of new pharmaceuticals with adalimumab, the first fully human mAb developed based on this method, to receive FDA approval in 2002 for rheumatoid arthritis, psoriasis and inflammatory bowel diseases. They both shared half of the 2018 Nobel Prize in chemistry with Frances Arnold. Since then, several mAbs have proven to be an effective therapy for almost every tumor type, starting with iNHLs where the anti-CD20 mAb rituximab, the first cancer immunotherapy, was initially FDA-approved three decades ago [2] (Table 1).

**Table 1:** Monoclonal antibody-based therapy for patients with i-NHL. Total sample is total number of patients involved in the study. PFS is recorded as rate or median. CALGB: cancer and leukemia group B, EFS: event free survival, FL: follicular lymphoma, mo: months, yr: years, MALT: mucosa-associated lymphoid tissue, MZL: marginal zone lymphoma, MM: multiple myeloma, MCL: mantle cell lymphoma, RT: Richter's transformation, SLL: small lymphocytic lymphoma, SMZL: splenic marginal zone lymphoma, WM: Waldenstrom's macroglobulinemia, BL: Burkitt's lymphoma, LPL: lymphoplasmacytic lymphoma, PMBCL: primary mediastinal B cell lymphoma, GZL: gray zone lymphoma, PTLD: post-transplant lymphoproliferative disorder, PBL: plasmablastic lymphoma, Benda: bendamustine, Chlor: chlorambucil, R: rituximab, O: obinutuzumab, Obs: observation, PoV: polatuzumab vedotin, PiV: pinatuzumab vedotin. NA = not available, NR = not reached.

Target	Study (Phase)	Population	Sample	ORR (%)	CR (%)	PFS
**CD20**						
	PRIMA: R-chemo +/- maintenance 10-yr follow up (III) [6]	FL	309 (R) vs 298 (Observation) (*N* = 607)	–	–	Median: 10.5 vs 4.1 yr (*P* < 0.001)
	RELEVANCE: R-Lenalidomide vs -chemo (III) [9]	Advanced FL	513 vs 517 (*N* = 1030)	61 vs 65	48 vs 53	Rate: 77 vs 78 at 3 yr (*p* = 0.48)
	AUGMENT: R- Lenalidomide vs –placebo (III) [10]	R/R indolent FL and MZL	178 vs 180 (*N* = 358)	78 vs 53	34 vs 18	Median: 39.4 vs 14.1 mo (*P* < 0.001)
	BRISMA/IELSG36: R-Benda in splenic MZL(II) [12]	Splenic MZL	*N* = 45	82	73	Rate: 90% at 3 yr
	GALLIUM: O-chemo vs R-chemo(III) [14]	FL	601 vs 601 (*N* = 1202)	88 vs 86	19 vs 23	Rate: 80 vs 73 at 3 yr (*P* = 0.001)
	GADOLIN: O-Benda vs Benda(III) [13]	R/R iNHL (FL, MZL, SLL, WM)	204 vs 209 (*N* = 413)	79 vs 77	17 vs 17	Median: 25.8 vs 14.1 mo (*P* < 0.001)
	HOMER: Ofatumumab vs Rituximab(III) [15]	R/R iNHL (FL, SLL, MZL, LPL)	219 vs 219 (*N* = 438)	50 vs 66	16 vs 20	Median: 16.3 vs 21.3 mo (*P* = 0.292)
	COMPLEMENTA/B: Ofatumumab-Benda Vs Benda(III) [16]	R/R i-NHL (SLL, MZL, LPL, FL)	172 vs 170 (*N* = 342)	73 vs 75	21 vs 17	Median: 16.7 vs 13.8 mo (*P* = 0.139)
	BRIGHT: R-Benda vs R-CHOP/CVP (III) [7]	I-NHL (LPL, MZL, MCL, FL)	213 vs 206 (*N* = 419)	97 vs 91	31 vs 25	Rate: 65.5 vs 55.8 at 5 yr [8] (*P* = 0.0025)
	STIL: R-Benda vs R-CHOP (III) [16]	I-NHL (FL, LPL, SLL, MZL, MCL)	261 vs 253 (*N* = 514)	93 vs 91 (NS)	40 vs 30	Median: 69.5 vs 31.2 mo (*P* < 0.0001)
	IELSG-19: R-chlor vs chlorvs R-monotherapy (III) [11]	MALT lymphoma	132 vs 131 vs 138 (*N* = 401)	94.7 vs 85.5 vs 78.3	78.8 vs 63.4 vs 55.8	Median: NR vs 8.3 yr vs 6.9 yr (p=0.0119) EFS: NR vs 5.1yr vs 5.6yr (p=0.0009)
	Ublituximab (I/II) [17]	R/R CLL, NHL (FL, MZL, MCL, DLBCL)	*N* = 35	45	13	Median: 7.7 mo
	Ublituximab + umbralisib (I/Ib) [18]	CLL/SLL and B-NHL (DLBCL, RT, MCL, FL, MZL)	*N* = 69	46	17	Median: 15.7 mo
**CD19**						
	Tafasitamab (II) [19]	R/R NHL (DLBCL, FL, MZL, CLL, MCL)	*N* = 92	29	8	Median: 8.8 mo (FL)
	Inebilizumab (I) [20]	R/R NHL (FL, DLBCL, CLL) and MM	*N* = 20	60	20	NA
	Loncastuximab tesirine (I) [39]	R/R NHL (DLBCL, MCL, FL, CLL, MZL)	*N* = 180	45.6	26.7	NA
**CD22**						
	CALGB50701: R-Epratuzumab (II) [23]	FL	*N* = 59	88.2	42.4	Median: 3.5 yr
	R-Epratuzumab (II) [24]	R/R NHL (FL, DLBCL, MZL, SLL, LPL)	*N* = 64	47	22	Median: 11 mo (FL)
**CD19/** **CD3**						
	Blinatumomab monotherapy (I) [26]	R/R NHL (FL, MCL, DLBCL, MZL)	*N* = 38	64	36	Median: 6.7 mo
	Blinatumomab + lenalidomide (I) [27]	R/R NHL (DLBCL, MCL, FL, MZL, BL, SLL)	*N* = 18	83	50	Median: 8.3 mo
**CD20/** **CD3**						
	Mosunetuzumab (I/Ib) [28]	R/R FL	*N* = 62	68	50	11.8 mo
	Glofitamab following obinutuzumab (I) [29]	R/R NHL (DLBCL, FL, PMBCL)	*N* = 171	53.8	36.8	Median: 11.8 mo (FL)
	Odronextamab (I) [30]	R/R NHL (DLBCL, FL, MCL, MZL)	28 (FL > 5 mg) (*N* = 127)	92.9 (FL)	75(FL)	Median: 12.8 mo
	Epcoritamab (I/II) [31]	R/R NHL (DLBCL, FL, MCL)	8 (FL) *N* = 67	100 (FL)	25 (FL)	NA
**PD-1**						
	CheckMate140: Nivolumab (II) [34]	R/R FL	*N* = 92	4	1	Median: 2.2 mo
	R-Pembrolizumab (II) [35]	R/R FL	*N* = 30	80	60	NA
**CD47**	*R* + 5F9 (Ib) [36]	R/R NHL (DLBCL, FL)	*N* = 22	50	436	NA
**CD30**	Brentuximab vedotin (II) [37]	R/R NHL (DLBCL, GZL, PMBL, FL, PTLD, PBL)	*N* = 48	44	17	3.9 mo
**CD79b**	ROMULUS: *R* + PoV or PiV (II) [38]	R/R NHL (DLBCL, FL)	41 (FL) *N* = 81	62 (R-PiV) 70 (R-PoV) (FL)	5 (R-PiV) 45 (R-PoV) (FL)	12.7 mo (R-PiV) 13.6 mo (R-PoV) FL

## 2 Targeting B-cells

### 2.1 First generation anti-CD20 monoclonal antibody (rituximab)

One of the most significant events in the timeline of mAb development was the identification of the CD20 transmembrane protein on B cells, as well as the discovery and production of the chimeric mAb rituximab. The CD20 protein is expressed on the surface of developing B-cells from early pre-B cells to later in differentiation, but is usually absent on plasma cells. Nadler, et al. published a serotherapeutic trial in 1980 with a patient who had a transient response to an antibody targeting CD20 [3]. Rituximab, initially labeled IDEC—C3B8, was developed as a type I anti-CD20 mAb that was FDA approved in 1997 for CD20+ *B*-cell relapsed or refractory (R/R) low grade or FL [4]. Rituximab mechanism of action is mainly through its Fc portion that mediates antibody-dependent cellular cytotoxicity (ADCC) and complement-dependent cytotoxicity (CDC) through natural killer cells leading to apoptotic cell death of CD20+ cells. Soon after its approval, rituximab became a cornerstone for the treatment of every B-cell malignancy in the frontline and R/R setting, as well as autoimmune disorders such as rheumatoid arthritis, vasculitis, and pemphigus vulgaris [5].

While patients with early stage FL can be potentially cured with radiation therapy, those with low tumor burden can have long term remission with single agent rituximab given weekly for four doses [4]. On the other hand, for patients with high tumor burden, as defined by the GELF criteria, the addition of rituximab to different chemotherapy backbones lead to improvement in overall response rate, progression free survival and overall survival [4]. As a relapse prevention strategy, the PRIMA study evaluated the benefit of every two months maintenance rituximab for two years following rituximab-based chemotherapy. The median progression free survival (PFS) was almost 11 years in the rituximab maintenance arm versus four years in the control arm [6]. However, this comes at the expense of increased risk of infection and financial toxicity.

Based on the BRIGHT and the STIL trials, Rituximab (R) in combination with bendamustine became a wildly adopted regimen in frontline iNHL given its relatively favorable toxicity profile compared to R-CHOP [7,8][16]. However, due to the adverse effects of cytotoxic chemotherapy, there has been increased interest in a chemo-free approaches for frontline and R/R FL (Table 1). RELEVANCE is the first phase 3 trial that evaluated a chemo-free regimen (R-lenalidomide) versus R-chemotherapy for induction therapy followed by maintenance rituximab [9]. At 120 weeks, both groups had similar CR, ORR, PFS and OS. However, there were more grade 3 or 4 neutropenia, febrile neutropenia, and infections in the R-chemotherapy arm, and more cutaneous reactions in the R-lenalidomide arm. While this trial did not meet its primary endpoint, the similar efficacy compared to standard chemo-immunotherapy, provides a proof of concept on the potential efficacy of mAb-based chemotherapy free approaches that might allow certain patients to avoid the side effects of chemotherapy in frontline FL. On the other hand, in the phase 3 AUGMENT study in R/R i-NHL, 12 cycles of R-Lenalidomide showed improved efficacy compared to R-placebo, although with increased expected toxicity [10]. This led to the 2019 FDA-approval of R-Lenalidomide in R/R FL and MZL.

*Rituximab* is also a major cornerstone in the treatment of MZL. While for limited stage MZL, radiation therapy or resection are the treatment of choice, weekly rituximab for four doses is the preferred option for splenic marginal zone lymphoma, advanced stage with low tumor burden, and in combination with chemotherapy (chlorambucil/bendamustine) for patients with high tumor burden [11][40]. In the IELSG-19, phase 3 trial of frontline MALT, patients were randomized to three arms: chlorambucil monotherapy, rituximab monotherapy, and the combination of rituximab and chlorambucil [11]. The primary endpoint was event free survival: chlorambucil at 5.1 years, rituximab at 5.6 years, while not reached for the combinations of rituximab and chlorambucil. Overall, the treatment was well tolerated in the three treatment arms. Furthermore, in the phase 2 BRISMA/IELSG36 trial, 45 patients with frontline splenic MZL were treated with R-Bendamustine, with an ORR of 82%, CR 73%, and a 3-year PFS of 90% [12]. Bendamustine can increase the risk of infections especially in the elderly. Given the efficacy and tolerability of rituximab-based therapy, current NCCN guidelines advise against splenectomy as the preferred first treatment modality for SMZL.

While rituximab is the first and oldest of the mAbs used in lymphoma therapies, its clinical development continues three decades later. In fact, several rituximab bio-similars have recently received FDA-approval including a subcutaneous form, rituximab hyaluronidase. This allows for cost reduction and less chair time, thus enhancing the treatment experience of patients.

### 2.2 Next generation anti-CD20 mAb (obinutuzumab, ofatumumab, and ublituximab)

Given the great success of rituximab in the treatment of B-cell malignancies, there has long been an interest in developing next generations of anti-CD20 mAbs with improved efficacy and tolerability. As rituximab is a chimeric mAb, second and third generation anti-CD20 agents used fully humanized mAbs that have enhanced binding affinity to CD20 with greater ADCC.

*Obinutuzumab (Gazyva)* is an anti-CD20 glycoengineered type II, IgG2 class monoclonal antibody with greater ADCC than Rituxan. It was first approved in 2013 in combination with chlorambucil for frontline CLL and in 2016, in combination with bendamustine followed by obinutuzumab (O) maintenance every two months for two years in patients with R/R FL that are refractory to rituximab based on the GADOLIN study [13]. For patients that are rituximab refractory, the GADOLIN trial, randomized patients to bendamustine single agent for 6 cycles with no maintenance versus 6 cycles of O-bendamustine followed by obinutuzumab maintenance every two months for two years. The obinutuzumab arm demonstrated improved PFS (25.8 months vs 14.1 months, *p* < 0.001) and overall survival (OS) (HR 0.67, *p* = 0.027), but at the expenses of increased grade 3–5 adverse events mainly hematological and infusion reactions. Of note, the clinical benefit was derived from the maintenance phase given similar ORR and PFS at end of induction in the two arms [13]. In 2017, obinutuzumab was approved in combination with bendamustine followed by obinutuzumab maintenance every two months for two years in patients with untreated FL based on the GALLIUM trial [14]. The GALLIUM study is a phase III trial that compared O-chemotherapy (bendamustine, CHOP, CVP) vs R-chemotherapy in patients with advanced FL. O-chemotherapy with O-maintenance had met its primary endpoint with improvement in the three years PFS (table 1) . However, grade 3–5 adverse events were more common in the O-arm 75% compared to the R-arm 69% [14].

*Ofatumumab (Arzerra)* is a type I fully human mAb that binds to a distinct epitope of the CD20 glycoprotein than rituximab with preclinical activity against B-cell malignancies with low CD20 and high CD55 and CD59 expression that are observed post rituximab. The HOMER trial is a phase III study in patients with R/R i-NHL following prior rituximab-based therapy including single agent rituximab. Patients were randomized to single agent ofatumumab vs rituximab. The study was closed early for futility as superiority was seen in the rituximab arm with an ORR of 66% compared to 50% in the ofatumumab arm (*P* = 0.0011) [15]. Similarly, the combination of bendamustine with ofatumumab showed no end of induction clinical benefit compared with single agent bendamutine in rituximab-refractory iNHL, but improvement in PFS with median of 16.7 vs 13.8 months due to the maintenance arm [16].

*Ublituximab* is a type I, IgG1 chimeric anti-CD20 mAb glycoengineered to have low fucose content oligosaccharides in the Fc region leading to higher ADCC. In vitro, ublituximab had a more effective ADCC response against B cells compared to rituximab and ofatumumab. In a phase I/II trial that included thirty-five patients with iNHL, ublituximab achieved an ORR of 42% in FL and 71% in MZL patients including two patients achieving CR in each subtype [17]. Combination therapies are currently in progress including the U2 regimen, ublituximab with umbralisib, a PI3K-*δ* and casein kinase-1ε inhibitor, in R/R NHL and CLL. In patients with FL and MZL, this regimen showed an ORR of 44% and 100% respectively [18]. The lower rates of immune-mediated toxicities is a distinctive feature of this regimen compared to other PI3K- *δ* inhibitors-based regimens.

### 2.3 Anti-CD19: tafasitamab, inebilizumab

CD19 is a transmembrane glycoprotein typically expressed in all B-cells and acts as a central positive regulatory response. Currently approved chimeric antigen receptor CAR-T cell therapies target CD-19 on B-cell acute lymphoblastic leukemia (ALL), diffuse large B cell lymphoma (DLBCL), and mantle cell lymphoma (MCL). There has been strong interest in developing CD19 mAbs for R/R NHL.

*Tafasitamab (MOR208)* is a humanized mAb that was studied in a phase II trial in patients with R/R B-NHL that have progressed following R-based therapy. An overall response rate of 29% including FL and MZL was observed with an equal efficacy in rituximab-refractory and non-refractory disease [19]. Those who had baseline peripheral NK cell above the threshold of 100 cells/µl had longer progression free survival likely reflective of a more robust ADCC. *Tafasitamab* in combination with lenalidomide is currently approved in R/R DLBCL. Furthermore, the phase 3 trial (InMIND) is evaluataing Tafasitamab plus Lenalidomide and Rituximab compared to Placebo plus Lenalidomide and Rituximab in patients with R/R FL or MZL (NCT04680052).

*Inebilizumab (MEDI551)* is another humanized anti-CD19 mAb currently in phase II in DLBCL (NCT01453205). A multicenter phase I study in Japan studied inebilizumab in patients with R/R NHL and MM. The ORR was 60% in the entire cohort, 82% in FL patients with a CR rate of 27% [20].

### 2.4 Anti-CD22: epratuzumab

CD22 is a transmembrane glycoprotein that is specific to B cells with higher concentrations in mature IgM+, IgD+ *B* cells that are present in FL and MZL. CD22 acts as a regulator of cell function and proliferation. LL2, a murine monoclonal antibody, was initially developed against Raji Burkitt lymphoma cells. It was found to induce phosphorylation of CD22 leading to ADCC. This lead to the development of epratuzumab, a humanized anti-CD22 mAb [21]. In the initial phase I/II clinical trial that included eight patients with FL and MZL, epratuzumab exhibited no single agent activity in these two subtypes [22]. In another phase II study with previously untreated FL, majority of them with stage IV, 59 patients received an extended induction with rituximab and epratuzumab over 9 months. The combination showed an impressive ORR of 88% and CR of 42%, but median PFS of only 3.5 years [23]. In R/R NHL, a phase II study of R-epratuzumab showed an expected lower activity including in patients with MZL [24].

## 3 Targeting T-cells

Immune evasion is a major hallmark of cancer. Therefore, activation of the patient's own immune system particularly cytotoxic T cells has shown impressive clinical activity especially in lymphoid malignancies. The basis of BiTE (bispecific T-cell engager) therapy is the generation of bispecific antibodies using protein engineering techniques to link two antigen binding domains, that will lead to the recruitment of cytotoxic T cells via CD3 to facilitate cell death of malignant B cells [25].

### 3.1 Anti-CD19/CD3: blinatumomab

CD19 is present on premature and mature B cells in NHL. Blinatumomab (Blincyto) was developed as a first-in-class bispecific antibody currently FDA-approved in R/R B-ALL since 2014. It consists of murine single chain variable fragments of anti-CD19 and anti-CD3 jointed by a glycine‑serine linker. In a long-term follow-up of MT103–104 phase 1 dose-escalation of blinatumomab monotherapy in R/R NHL, significant improvement in response and OS was observed for patients who received ≥60 mg/m^2^, the maximum tolerated dose, without evidence of long-term toxicities, especially neurocognitive impairments [26]. Blinatumomab in combination with lenalidomide was studied in a small phase I trial in patients with R/R NHL. The combination was found to be safe, effective with encouraging activity with ORR and CR of 83% and 50% [27].

### 3.2 Anti-CD20/CD3: mosunetuzumab, glofitamab, odronextamab, epcoritamab

A number of other bispecific mAbs are currently under development in early-stage trials. Four major bispecific antibodies combining anti-CD20 and anti-CD3 in varying ways have reported promising clinical activity.

Mosunetuzumab *(RG7828, RO7030816)* uses a humanized IgG1 base full-length bispecific CD20/CD3 antibody. Mosunetuzumab was evaluated as a single agent in a large phase I/Ib study in patients with R/R NHL including those refractory to CAR-T therapy, of whom 11 patients with FL. Of the four evaluable FL patients with prior CAR-T therapy, all responded with 2 CR. In iNHL patients, the ORR was 68% with a CR rate of 50% [28]. In July 2020, Mosunetuzumab was granted Breakthrough Therapy Designation by the FDA, for the treatment of adult patients with relapsed or refractory follicular lymphoma. Furthermore, Mosunetuzumab is being evaluated in subcutaneous form to reduce the risk of cytokine release syndrome (CRS) and in frontline DLBCL.

*Glofitamab* has a similar base with bivalent CD20 and monovalent CD3, described as a 2:1 molecular configuration with two Fab regions for CD20 and one Fab region for CD3. In a phase I trial, patients were pretreated with obinutuzumab, prior to glofitamab infusion. All iNHL were of FL, with an ORR of 77% [29]. Glofitamab is being further evaluated in combination, and in the frontline setting.

*Odronextamab (REGN1979)* is a hinge-stabilized, fully humanized IgG4-based bispecific CD20/CD3 antibody. In a phase I study of R/R NHL including post CAR-T cell therapy, 28 evaluable patients with FL were treated with single agent odronextamab. There was significant activity observed with an ORR of 93% and CR of 75% that were seen at doses ≥ 5 mg^30^.

*Epcoritamab (GEN3013)* is also a humanized IgG base monovalent CD20 and CD3 with an important advantage of being administered subcutaneously. Preliminary data of a phase I/II trial in heavily pre-treated patients including post CAR-T cell therapy, single agent epcoritamab showed an excellent ORR of 100% among all eight patients with R/R FL and a complete response in four of them [31].

With these promising results in early phase trials, BiTE therapy may be a cost effective and more tolerated alternative to patients that are candidates for chimeric antigen-receptor (CAR) T-cell therapy. CAR-T cell has a similar concept to BiTE, given that the patient's own T-cells are genetically modified to target specific markers such as CD-19 present on the hematologic cancer cells. Interestingly, CAR-T cell process is complex, and lengthy given the manufacturing time that takes several weeks. Therefore, BiTE therapy may provide an “off the shelf alternative”. However, both therapies are usually associated with risk of cytokine release syndrome and neurocognitive toxicities that require close clinical monitoring.

### 3.3 Anti-PD-1: nivolumab, pembrolizumab

Programmed cell death protein 1 (PD-1) ligand expression is a major mechanism of tumor evasion from the patient's own immune surveillance. In neoplastic cells and its tumor microenvironment, PD-1 L binds to PD-1 leading to inhibitory signals of cytotoxic T cells and their exhaustion. The success of blocking the PD-1/PD1-L axis has revolutionized cancer therapy with approval of several PD-1/PD-1 L inhibitors across several tumors, including tumor agnostic indications. Genetic basis for immune evasion via upregulation of PD-1 L have been identified in classical Hodgkin's lymphoma (cHL) and/or primary mediastinal B-cell lymphomas (PMBCL) [32]. Two PD-1 inhibitors have reported impressive activity and are currently approved for these two lymphoma subtypes: nivolumab (Opdivo) and pembrolizumab (Keytruda). They are fully humanized IgG4 mAbs that block the binding of PD-1 L or 2 L – PD-1 on T cells [33].

In FL, PD-1 T-cells are also present; however, T cells that infiltrate FL tumors have higher PD-1 expression, a marker of exhaustion, compared to peripheral T cells. There are also numerous PD-1 T cells in MZL. Interestingly, FL and MZL cells express low rates of PD-1 L at 5% and 10%, respectively. The CheckMate 140 study examined 92 patients with R/R FL treated with nivolumab monotherapy. However, there was virtually no response [34]. Alternatively, pembrolizumab has had more success in combination with rituximab. Preliminary results from a phase II study showed an ORR of 80% with a CR of 60% in 27 patients with FL [35]. Although monotherapy is unlikely to be effective alone, combination strategies of PD-1/PDL-1 inhibitors with standard and/or novel therapies may broaden the benefit from this class of mAb in the treatment of iNHLs.

## 4 Targeting macrophages and tumor microenvironment

### 4.1 Anti-CD47: Hu5F9-G4

CD47 is an anti-phagocytic “don't eat me” signal expressed by almost all cancer cells to evade the immune system by binding to the signal regulatory protein alpha (SIRP*α*) located on phagocytes, including macrophages. Overexpression of CD47 is an independent predictor of poor prognosis in patients with malignancy [36].

*Hu5F9-G4 (5F9)* is a humanized anti-CD47 mAb therapy to augment phagocytosis of tumor cells and induce T-cell response by cross-presentation of tumor antigens by macrophages. This is done selectively by unmasking pro-phagocytic signals expressed on tumor cells rather than normal cells. A phase Ib study involved 22 patients with R/R NHL treated with 5F9 and rituximab. Transient anemia was an adverse effect due to 5F9s activity of blocking CD47 on aged red blood cells. However, only minimal evidence of hemolysis was observed and patients typically appropriately responded. Out of the 7 patients with FL, the overall response was seen in 5 with 3 patients achieving a CR. Median duration of response was not reached at cutoff of 8 months [36]. This provides a proof-of-concept on the therapeutic potential for developing mAbs targeting phagocytes.

## 5 Targeted chemotherapy

### 5.1 Antibody drug conjugates: brentuximab vedotin, polatuzumab vedotin, loncastuximab tesirine

Antibody drug conjugates (ADC) consist of a mAb linked to a payload with the intent for targeted chemotherapy delivered precisely to the tumor bearing the corresponding receptor, reflecting the concept of the “magic bullet” formulated by Paul Ehrlich in the early twentieth century. Cytotoxic payloads typically include antimitotic and DNA binding agents. Brentuximab vedotin (BV) and polatuzumab vedotin (PoV), loncastuximb (Lonca) are the three currently FDA-approved ADCs in lymphomas without an indication for iNHL. BV is an anti-CD30 antibody with monomethyl auristatin E (MMAE) payload approved in classic Hodgkin lymphoma, cutaneous and peripheral T cell lymphomas. A phase II trial in patients with R/R NHL showed a 44% ORR in non-diffuse large B cell lymphoma NHL with a 17% CR [37]. PoV is an anti-CD79b mAb conjugated with MMAE, current approved in combination with BR in R/R DLBCL. The ROMULUS trial evaluated two anti-CD79b ADC, PoV and pinatuzumab vedotin (PiV) in combination with rituximab in patients with R/R NHL [38]. Out of the 41 patients with FL, responses to PiV and PoV were 62 and 70%, respectively. More CR were seen with PoV 45% compared with 5% in PiV [38]. Loncastuximab tesirine (ADCT-402) is a humanized anti-CD19 mAb conjugated to a pyrrolobenzodiazepine dimer toxin, currently approved as a single agent in R/R DLBCL. In a large phase 1 trial that included R/R i-NHL, loncastuximab showed an excellent single agent activity with an ORR 46% and CR 27% [39]. It is currently being further developed in combination therapy and in the frontline setting.

## 6 Conclusion

This review highlights the major progressions in the use of mAbs over the past 30 years. Although FL and MZL are indolent in nature, but they remain incurable. Advanced stage and more aggressive disease tempo of these lymphomas are common. mAb therapy especially anti-CD20 has been a crucial step forward in reducing disease burden and prolonging overall survival when combined with chemotherapy. Furthermore, given their specificity, favorable therapeutic index, mAbs are well poised to offer an alternative to conventional cytotoxic agents. Finally, with current advances in novel antigen identification, antibody formation, and improvement in our bio-engineering capabilities, it is no surprise that we will continue to witness an expansion of these various therapies and their combination strategies until i-NHL becomes curable.
